# Safety and effectiveness of carotegrast methyl in patients with ulcerative colitis: an interim report of post-marketing surveillance in Japan

**DOI:** 10.1093/crocol/otag105

**Published:** 2026-09-18

**Authors:** Masayuki Saruta, Yoshiharu Miura, Mie Iwaida, Minami Umeyama, Shinsuke Kurosu, Kiyotoshi Kuramoto, Kenji Watanabe

**Affiliations:** Department of Internal Medicine, Division of Gastroenterology and Hepatology, The Jikei University School of Medicine, Tokyo, Japan; Department of Neurology, Tokyo Metropolitan Cancer and Infectious Diseases Center Komagome Hospital, Tokyo, Japan; Medical Department, EA Pharma Co., Ltd, Tokyo, Japan; Medical Department, EA Pharma Co., Ltd, Tokyo, Japan; Pharmaceutical Development Department, EA Pharma Co., Ltd, Tokyo, Japan; Department of Post-Marketing Surveillance Pharmacovigilance & Post-Marketing Surveillance, Kissei Pharmaceutical Co., Ltd, Tokyo, Japan; Internal Medicine for Inflammatory Bowel Disease, Toyama University Hospital, Toyama, Japan

**Keywords:** carotegrast methyl, safety and effectiveness, post-marketing surveillance, interim report, ulcerative colitis

## Abstract

**Background:**

The first multicenter, prospective post-marketing surveillance (PMS) in Japan was conducted to confirm the safety and effectiveness of carotegrast methyl (hereafter referred to as carotegrast) in the routine clinical practice for ulcerative colitis (UC) in Japan. Here, we report the results of the interim analysis of PMS.

**Methods:**

The ongoing PMS was initiated in June 2022 and will be continued until May 2028. Study patients are individuals with moderately active UC who were refractory or intolerant to 5-aminosalicylic acid, and who started carotegrast therapy for the first time. An interim analysis was conducted based on the data cut-off (March 27, 2025).

**Results:**

Safety and effectiveness analysis sets included 296 and 290 patients, respectively. In the safety analysis set, 36.8% of patients received carotegrast for <8 weeks and 63.2% for ≥8 weeks. The most common reason for treatment discontinuation was inadequate effectiveness in 44.0% of patients who received carotegrast for <8 weeks, and symptom improvement in 66.8% for ≥8 weeks. Adverse drug reactions (ADRs) were reported in 25 patients (8.45%). No serious ADRs, ADRs relevant to progressive multifocal leukoencephalopathy, and central nervous system-related ADRs were reported. No pregnancies were reported. One patient (0.34%) experienced an ADR of pyelonephritis related to infections. The proportions of patients with clinical response, clinical remission and rectal bleeding disappearance (defined by using partial Mayo score) were 146/185 (78.9%), 133/189 (70.4%) and 137/190 (72.1%) after 8 weeks carotegrast administration, respectively.

**Conclusions:**

No new safety and effectiveness concerns were identified in the interim analysis for PMS.

## Introduction

Ulcerative colitis (UC) is a chronic inflammatory bowel disease (IBD) that affects the rectum and colon to varying degrees.^1^ UC impairs patients’ quality of life secondary to inflammation in the large bowel causing chronic diarrhea and rectal bleeding, and patients with UC have increased risks for rectal/colorectal resection and cancer.^2^

Recently, the UC therapeutic landscape is expanding with the development of molecularly targeted agents, including oral small molecules and injectable biologics.^1–3^ Carotegrast methyl (CAROGRA®, hereafter referred to as carotegrast) is a newly developed small molecule of α4-integrin antagonist that received its first approval in March 2022 for the treatment of UC in Japan.^4^ The Japanese treatment guidelines for UC recommend its use as induction therapy for patients with moderately active UC who are refractory or intolerant to 5-aminosalicylic acid (5-ASA).^5^ According to the approved prescribing information in Japan, carotegrast methyl is generally administered orally at a dose of 960 mg 3 times daily for 8 weeks, and treatment may be continued for up to 6 months when additional therapeutic benefit is expected. Treatment discontinuation should be considered when remission is achieved during the treatment period.

Oral carotegrast prevents the development of experimental colitis in mice,^6^ and its efficacy and safety have been shown in a randomized, double-blind, placebo-controlled, phase 2a study.^7^ A multicenter, randomized, double-blind, placebo-controlled, phase 3 study of carotegrast has further demonstrated its efficacy and safety in patients with moderately active UC who had an inadequate response or intolerance to 5-ASA.^8^ The study reveals that a significantly higher proportion of patients achieved clinical response defined with a reduction of Mayo score in the carotegrast group (45%, 46/102 patients) compared to the placebo group (21%, 21/101 patients) at week 8. During the 8-week treatment and 16-week extension treatment periods, the most common treatment-related adverse event (AE) was nasopharyngitis in the carotegrast (incidence: 3%) and placebo group (4%), and most AEs were mild-to-moderate in the severity.

It is important to confirm the safety and effectiveness of a launched drug, and investigate an appropriate use of the drug in real-world clinical settings, since the available evidence only from clinical trials is still limited, especially in patient populations with more various background factors. There is limited evidence that assess the safety and effectiveness of carotegrast in real-world clinical setting after its launch in Japan (May 2022). Additionally, carotegrast treatment has concerns that may increase the risk of progressive multifocal leukoencephalopathy (PML), which is a serious adverse effect of the integrin α4 antibody natalizumab,^9^ although no PML has been reported in the previous phase 3 study of carotegrast.^8^

We are currently conducting a post-marketing surveillance (PMS) among patients with UC who were treated with carotegrast after its launch in Japan to confirm the safety and effectiveness of carotegrast in routine clinical practice. This article reports interim PMS analysis results based on the cutoff data collected through March 27, 2025.

## Methods

### Study design

This is an ongoing PMS for the carotegrast in Japan, which was initiated in June 2022 and will be continued until May 2028. The primary objective of this PMS is to evaluate the safety of carotegrast, and the aim is also to collect information on its effectiveness in patients with moderately active UC under routine medical use, and utilize information for the appropriate use of the drug in real-world clinical practice for UC. The PMS consists of 2 carotegrast treatment periods of first- and re-treatment phases (in principle 8 weeks, up to 6 months in both phases), followed by observation period of up to 52 weeks in each phase. During the observation period, safety-related information, subsequent treatments, and re-treatment with carotegrast methyl, when applicable, were collected. Prespecified effectiveness assessments, including partial Mayo score evaluations, were conducted during the treatment period and were not routinely scheduled during the post-treatment observation period. In routine clinical practice in Japan, treatment is generally administered for 8 weeks and may be continued for up to 6 months when additional therapeutic benefit is expected. Therefore, treatment duration may vary according to individual patient response and physician judgment. Treatment discontinuation is considered when remission is achieved in accordance with the approved prescribing information.

The longest total observation period is 3 years per patient after the start of first treatment with carotegrast. The target sample size is set to 300 patients to provide a power of ≥95% to detect at least 1 patient reporting an AE of infections at an incidence of 1.0%. The PMS was registered at the Japan Registry of Clinical Trials (jRCT2031220193).

Here, we report the results of the interim analysis of the PMS based on the cutoff data until March 27, 2025, for the first-treatment phase, excluding the follow-up observation period. This interim analysis was conducted as part of a scheduled periodic safety reporting process during the conduct of the PMS. A final analysis is planned after completion of patient follow-up and collection of the full PMS dataset.

### Ethical considerations

The PMS is being conducted in accordance with the ministerial ordinance on Good Post-marketing Study Practice in Japan. The survey protocol was reviewed and approved by the Pharmaceuticals and Medical Devices Agency prior to survey initiation. All participants provided informed consent before participating in the survey.

### Patients

The target patients for the PMS were individuals with moderately active UC who were refractory or intolerant to 5-ASA, and who started carotegrast therapy for the first time under routine medical use in any of the 132 medical institutions in Japan. Patients with informed consent to participate in the PMS were registered centrally, and their medical data (case report form: CRF) were obtained via a web-based electronic data capture system from the medical institutions. The patient registration period was from June 2022 to September 2024. Patients were required to be registered within 14 days from the first administration of carotegrast, with the first treatment day defined as Day 1.

### Study assessments

#### Patient characteristics

Demographic and baseline data included information on sex, age, height, weight, pregnancy (women only), duration of UC, disease type of UC, smoking habits, inadequate response to 5-ASA treatment or intolerance status, intolerance or resistance to steroids, complications, medical history, and previous and concomitant medications for UC. Previous and concomitant medications for UC were recorded based on treatments administered during the 3 months prior to the initiation of carotegrast.

#### Administration status of carotegrast

The status of carotegrast administration was monitored by recording the starting date, daily dose, discontinuation date, reasons for discontinuation, and reasons for continuation (if administration period was beyond 8 weeks).

#### Safety

Safety was assessed in the safety analysis set, which included all patients who received at least 1 dose of carotegrast, including those treated outside the approved indication in Japan. The endpoint for the safety assessment was incidences of adverse drug reactions (ADRs), defined as AEs that were considered to be related to carotegrast by the physicians. The seriousness of the observed ADRs was also assessed by the physicians according to the International Conference on Harmonization standards. The observed ADRs were tabulated by system organ class (SOC) and preferred term (PT) using Medical Dictionary for Regulatory Activities/Japanese (MedDRA/J) Version 27.1. Each SOC was tabulated as the number of patients who experienced events, and each PT was tabulated as the number of events. If events of the same PT occurred more than once in the same patient, they were counted as 1 event for tabulation purposes. An intensive investigation item referred to the incidence of central nervous system (CNS)-related ADRs, including hemiplegia, impairment of cognitive function, dyslalia, cerebellar ataxia symptom (dizziness and vertigo), aphasia, quadriplegia, vision abnormal, dysphagia, involuntary movement, and cranial nerve paralysis. Subgroup analyses for the incidence of ADRs were performed by stratifying into patient background factors.

#### Effectiveness

The proportions of patients with a clinical response, clinical remission and rectal bleeding disappearance were evaluated using the partial Mayo score and its subscore,^10^ as follows; A clinical response was defined as a reduction in the partial Mayo score from baseline of ≥25% and ≥2 points and reduction in rectal bleeding subscore from baseline of ≥1 point or rectal bleeding subscore ≤1 point. A clinical remission was defined as the partial Mayo score ≤2 points and all subscores ≤1 point. A rectal bleeding disappearance was defined as a rectal bleeding subscore of 0 point. The endoscopic assessment used to determine the baseline Mayo score was based on an endoscopic examination performed within 28 days before the initiation of carotegrast.

#### Other assessments

Medication adherence status and perception of symptom improvement were evaluated as patient reported outcomes (PROs) based on patient self-report. These assessments were study-specific items and were not based on validated PRO instruments. Medication adherence was assessed using 5 predefined categories based on the proportion of prescribed doses taken: 100%, ≥80%–<100%, ≥50%–<80%, ≥20%–<50%, and <20%. Patients’ perception of symptom improvement was assessed using a binary response (“perceived improvement” or “no perceived improvement”). Assessments were performed at the same scheduled time points as the effectiveness assessments. When a patient reported perceived improvement, subsequent assessments of this outcome were discontinued. Missing responses were not imputed, and analyses were based on available data.

### Statistical analysis

The safety analysis set consisted of all patients who received carotegrast at least once and had available safety data. The effectiveness analysis set included patients in the safety analysis set, excluding those for whom effectiveness had not been assessed (ie, without at least 1 post-baseline effectiveness assessment) and those who were not treated with carotegrast within the condition of its approved dosage and administration in Japan. Changes in the partial Mayo score from baseline were analyzed using the Wilcoxon signed-rank test. All statistical tests were 2-sided and a significance level of 5% was used. No missing values were imputed in the primary analysis. To assess the robustness of the results to missing partial Mayo score assessments, a sensitivity analysis using last observation carried forward (LOCF) was performed. For patients with missing post-baseline partial Mayo scores at any assessment time point, the most recent available post-baseline partial Mayo score was carried forward to the subsequent assessment time point(s), and changes from baseline were evaluated using the Wilcoxon signed-rank test. In addition, week-8 effectiveness endpoints (clinical response, clinical remission, and rectal bleeding disappearance) were evaluated in a sensitivity analysis by imputing missing week-8 partial Mayo score components (stool frequency, rectal bleeding, physician’s global assessment) using LOCF and applying the pre-specified endpoint definitions.

All analyses were performed using SAS version 9.4 for Windows. This interim analysis was undertaken to support a scheduled periodic safety reporting activity required during the conduct of the PMS. The analysis plan was finalized prior to data extraction and analysis.

## Results

### Patient disposition

A total of 434 patients with UC were registered in the PMS (Figure 1). The CRF was collected from 302 patients who completed the first-treatment phase (not including the follow-up observation period). Of them, 296 patients were eligible for the safety analysis set after excluding 6 patients with no hospital visits after the first time, no carotegrast intake, unlocked data in the CRF, and registration at ≥15 days after carotegrast prescription. Among the safety analysis set, the effectiveness analysis set included 290 patients after excluding 6 patients with deviations from the approved dosage or administration period of carotegrast in Japan.

**Figure 1:**
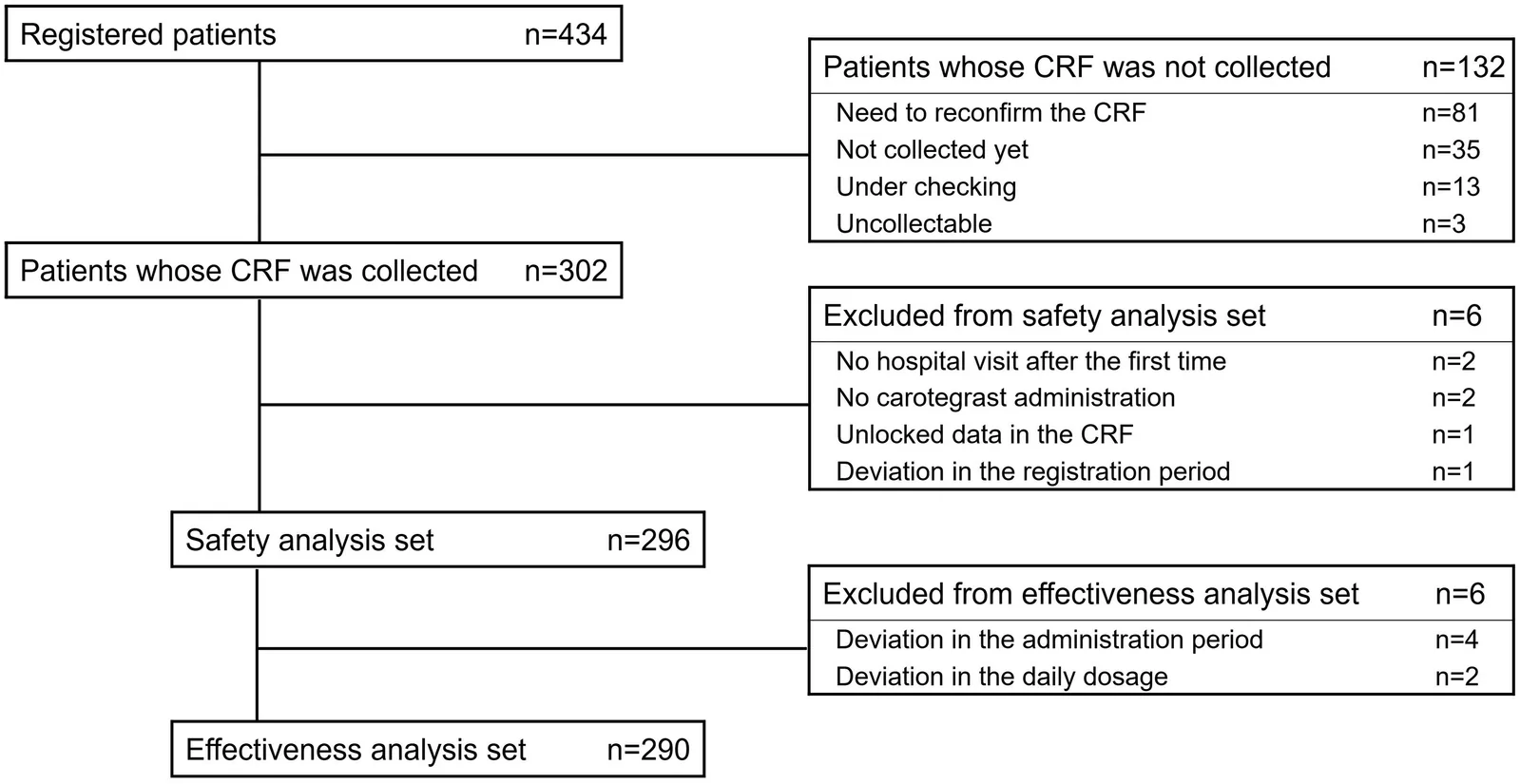
Patient disposition for this interim analysis of the PMS. CRF, case report form; PMS, post-marketing surveillance.

### Patient characteristics

In the safety analysis set, the mean (±SD) age of the patients was 43.2 ± 16.1 years, and 11.5% were aged ≥65 years (Table 1). Males accounted for 56.1% of the patients. The duration of UC was <1 year in 14.2%, 1 to <5 years in 33.4% and ≥5 years in 51.7% of patients. More than half (58.4%) of patients had pancolitis, 33.4% had left-sided colitis, and 8.1% had proctitis. Regarding previous medication, the majority (91.6%) of patients had an inadequate response to 5-ASA, and 8.4% were intolerant to 5-ASA. Oral 5-ASA was most commonly used in 87.2% of the patients, followed by 5-ASA enema and suppository in 13.2%, oral steroids in 7.1%, steroids enema and suppository in 18.6%, and intravenous corticosteroids in 0.7%.

**Table 1:** Patient characteristics.

Characteristics	Statistics/*n* (%)
**Total**	296
**Sex**	
** Male**	166 (56.1)
** Female**	130 (43.9)
**Age, years**	
** Mean ± SD**	43.2 ± 16.1
** <15**	0 (0.0)
** ≥15 to <65**	262 (88.5)
** ≥65**	34 (11.5)
**Weight, kg**	
** Mean ± SD**	60.49 ± 11.99^a^
**BMI, kg/m^2^**	
** Mean ± SD**	22.23 ± 3.64^b^
**Duration of UC, years**	
** <1**	42 (14.2)
** ≥1 to <5**	99 (33.4)
** ≥5**	153 (51.7)
** Unknown**	2 (0.7)
**Disease type of UC**	
** Pancolitis**	173 (58.4)
** Left-sided colitis**	99 (33.4)
** Proctitis**	24 (8.1)
**Response status to treatment with 5-ASA**	
** Inadequate response**	271 (91.6)
** Intolerance**	25 (8.4)
**Smoking habits**	
** Never**	266 (89.9)
** Current/former**	29 (9.8)
** Unknown**	1 (0.3)
**Baseline Mayo score^c^**	
** Mean ± SD**	6.7 ± 1.9
** 0-2**	1 (0.9)
** 3-5**	32 (27.6)
** 6-10**	83 (71.6)
** 11-12**	0 (0.0)
**Baseline partial Mayo score^d^**	
** Mean ± SD**	4.8 ± 1.6
** 0-4**	119 (41.8)
** 5-7**	159 (55.8)
** 8-9**	7 (2.5)
**Prior treatment drugs for UC**	
** None**	26 (8.8)
** Yes**	270 (91.2)
** 5-ASA**	
** Total**	259 (87.5)
** Oral**	258 (87.2)
** Enema and suppository**	39 (13.2)
** Steroids**	
** Total**	75 (25.3)
** Enema and suppository**	55 (18.6)
** Oral**	21 (7.1)
** Oral budesonide**	3 (1.0)
** Intravenous corticosteroids**	2 (0.7)
** Immunomodulators**	17 (5.7)
** Biologics**	3 (1.0)
** JAK inhibitors**	1 (0.3)
** Calcineurin inhibitors**	0 (0.0)
**Concomitant drugs for UC**	
** None**	22 (7.4)
** Yes**	274 (92.6)
** 5-ASA**	
** Total**	262 (88.5)
** Oral**	261 (88.2)
** Enema and suppository**	36 (12.2)
** Steroids**	
** Total**	53 (17.9)
** Enema and suppository**	43 (14.5)
** Oral**	12 (4.1)
** Intravenous corticosteroids**	1 (0.3)
** Oral budesonide**	0 (0.0)
** Immunomodulators**	11 (3.7)
** Biologics**	1 (0.3)
** JAK inhibitors**	0 (0.0)
** Calcineurin inhibitors**	0 (0.0)

Abbreviations: 5-ASA, 5-aminosalicylates; BMI, body mass index; JAK inhibitors, Janus Kinase inhibitors; UC, ulcerative colitis.

a *N* = 242 as the denominator.

b *N* = 228 as the denominator.

c *N* = 116 as the denominator.

d *N* = 285 as the denominator.

### Administration status of carotegrast

In the safety analysis set, 36.8% of patients received carotegrast for <8 weeks and 63.2% for ≥8 weeks in the first-treatment phase (Figure 2). The most common duration of carotegrast administration was ≥16–≤26 weeks in 26.4% of patients, followed by ≥8 weeks to <12 weeks in 23.6% and ≥4 weeks to <8 weeks in 20.3%. Four patients continued carotegrast administration for >6 months (26 weeks). The mean (±SD) duration of carotegrast administration was 82.3 ± 52.0 days in the total safety analysis set, 33.1 ± 18.4 days in patients who received carotegrast for <8 weeks, and 111.0 ± 42.9 days in patients who received it for ≥8 weeks. In almost all of the patients (99.3%), the daily dosage of carotegrast during the first-treatment phase was the approved dosage of 960 mg 3 times per day.

**Figure 2:**
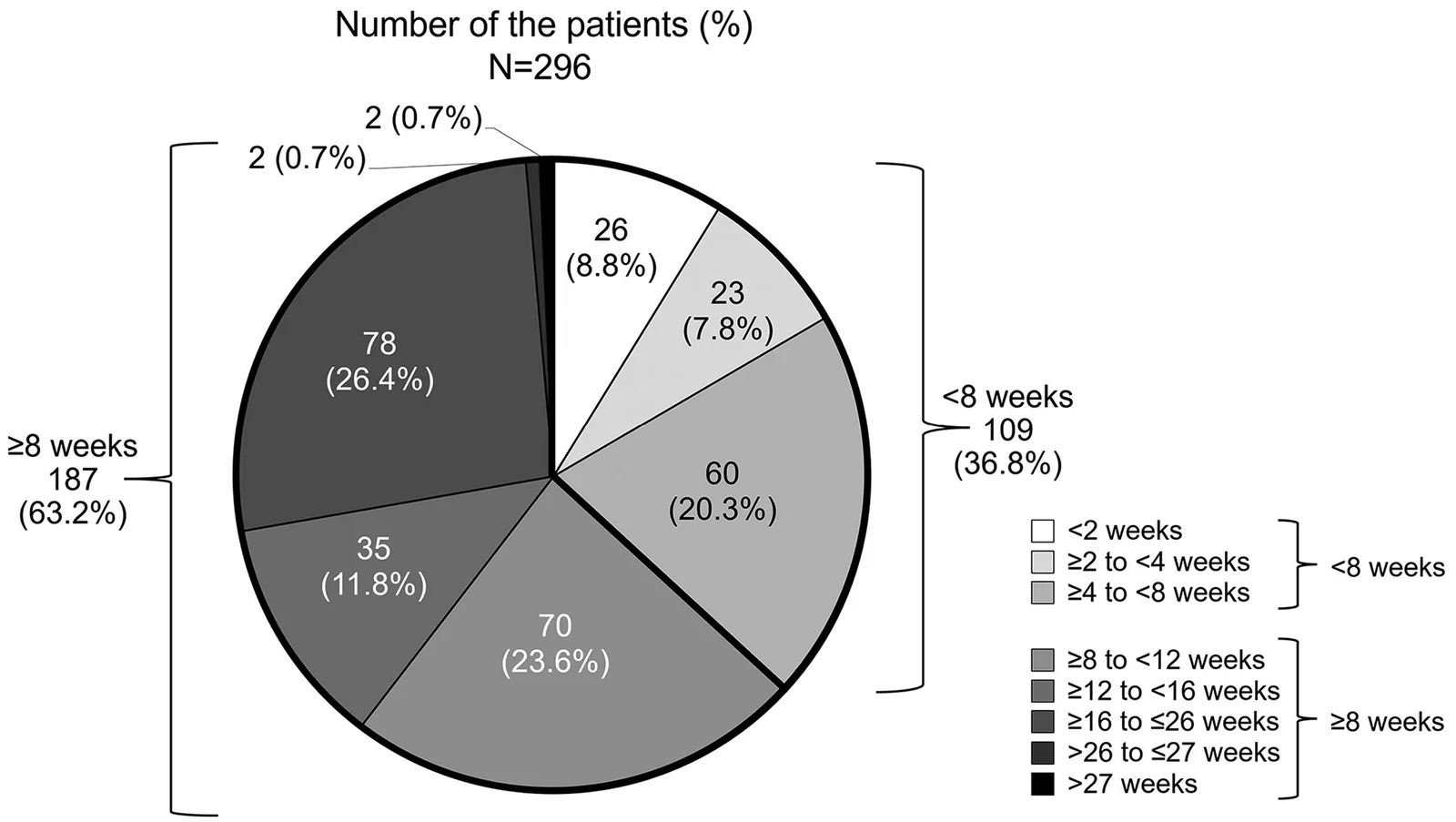
Duration of carotegrast administration.

The reasons for treatment discontinuation included symptom improvement (54.1%), inadequate effectiveness (32.4%) and occurrence of AEs (8.1%) in the total safety analysis set (Table 2). The most common reason was inadequate effectiveness in 44.0% of patients who received carotegrast for <8 weeks, and symptom improvement in 66.8% of patients who received it for ≥8 weeks.

**Table 2:** Reasons for treatment discontinuation.

Reasons *n* (%)	Duration of carotegrast administration		
	Total (*N* = 296)	<8 weeks (*N* = 109)	≥8 weeks (*N* = 187)
**Symptom improvement**	160 (54.1)	35 (32.1)	125 (66.8)
**Inadequate effectiveness**	96 (32.4)	48 (44.0)	48 (25.7)
**Occurrence of AEs**	24 (8.1)	18 (16.5)	6 (3.2)
**Patient’s request (excluding AEs occurrence)**	4 (1.4)	4 (3.7)	0 (0.0)
**No visits after the first few visits**	4 (1.4)	2 (1.8)	2 (1.1)
**Hospital transfer**	3 (1.0)	2 (1.8)	1 (0.5)
**Others**	5 (1.7)	0 (0.0)	5 (2.7)

Abbreviation: AE, adverse event.

Among the 24 patients who discontinued carotegrast because of AEs, the reported events were nausea (*n* = 6), UC (*n* = 5), headache (*n* = 4), urticaria (*n* = 2), pyelonephritis (*n* = 1), interstitial lung disease (*n* = 1), upper abdominal pain (*n* = 1), cheilitis (*n* = 1), esophagitis (*n* = 1), appetite decreased (*n* = 1), eczema (*n* = 1), arthralgia (*n* = 1), and lymphocyte count increased (*n* = 1).

### Safety

In the safety analysis set, 43 patients (14.53%) experienced AEs, and ADRs were reported in 25 patients (8.45%) during the first-treatment period (Table 3). No serious ADRs were observed in any patients. No ADRs relevant to PML were reported. In addition, no pregnancies were reported during the study period. One patient (0.34%) experienced an ADR of pyelonephritis related to infections. One patient (0.34%) experienced an ADR of interstitial lung disease, which occurred 54 days after the start of carotegrast administration; the event was non-serious, and the outcome was recovered.

**Table 3:** Incidence of ADRs in the safety analysis set (*n* = 296).

ADRs	**Incidence**
SOC/PT	*n* (%)
**Any ADRs**	25 (8.45)
** Serious ADRs**	0 (0.0)

** Gastrointestinal disorders**	12 (4.05)
** Nausea**	8 (2.70)
** Abdominal distension**	1 (0.34)
** Abdominal pain upper**	1 (0.34)
** Cheilitis**	1 (0.34)
** Constipation**	1 (0.34)
** Nervous system disorders**	6 (2.03)
** Headache**	6 (2.03)
** Investigations**	4 (1.35)
** Eosinophil count increased**	3 (1.01)
** Lymphocyte count increased**	1 (0.34)
** Monocyte count increased**	1 (0.34)
** Skin and subcutaneous tissue disorders**	5 (1.69)
** Urticaria**	3 (1.01)
** Dermatitis atopic**	1 (0.34)
** Eczema**	1 (0.34)
** Infections and infestations**	1 (0.34)
** Pyelonephritis**	1 (0.34)
** Respiratory, thoracic, and mediastinal disorders**	1 (0.34)
** Interstitial lung disease**	1 (0.34)
** Musculoskeletal and connective tissue disorders**	1 (0.34)
** Arthralgia**	1 (0.34)

Translated from MedDRA/J version (27.1).

Abbreviations: ADR, adverse drug reaction; AE, adverse event; PT, preferred term; SOC, system organ class.

None of the patients experienced CNS-related ADRs, which were defined as intensive investigation items in the PMS. In 4 patients who continued carotegrast administration for >6 months, ADR of abdominal distension was observed in 1 patient, and ADR occurred 13 weeks after the start of administration. Among the 4 patients categorized as having an administration period of >6 months, 2 exceeded 6 months by only a few days based on the operational duration definition, whereas the remaining 2 patients had treatment interruption(s) and subsequent re-initiation, with the drug-free interval(s) included in the calculated administration period. Therefore, the reported duration should be interpreted in this context.

Subgroup analyses for the incidence of ADRs revealed that no ADRs occurred in patients aged ≥65 years (34 patients). However, given the small number of elderly patients (*n* = 34), the statistical power to detect even moderately frequent AEs was limited. Meanwhile, ADRs occurred in 6/119 patients (5.04%), 15/159 patients (9.43%) and 3/7 patients (42.9%) who had a baseline partial Mayo score of 0–4, 5–7 and 8–9, respectively. Because the partial Mayo score 8–9 subgroup comprised only 7 patients, this finding should be considered exploratory and interpreted with caution. In addition, given that carotegrast methyl is indicated for moderately active UC and is less frequently used in patients with more severe disease in routine clinical practice, the number of patients in this higher disease activity subgroup was inherently limited in this PMS.

Few patients (≤1 patient) had complications such as renal impairment, hepatic function abnormal and cholestasis at baseline, and no ADRs occurred in these patients.

In the subgroup of 14 patients who received concomitant immunomodulators, no additional safety signals were observed. ADRs were reported in 2 patients (nausea and headache); both events were non-serious, and the outcomes were recovered.

### Effectiveness

In the effectiveness analysis set, the change in partial Mayo score after the start of carotegrast administration is shown in Figure 3. The scores decreased over time from 2 weeks after the start of administration, with statistically significant improvements at all assessment time points compared to the baseline score. In a sensitivity analysis using LOCF to impute missing post-baseline partial Mayo score assessments at any time point, partial Mayo scores also showed statistically significant reductions from baseline at all assessed time points, consistent with the primary analysis.

**Figure 3:**
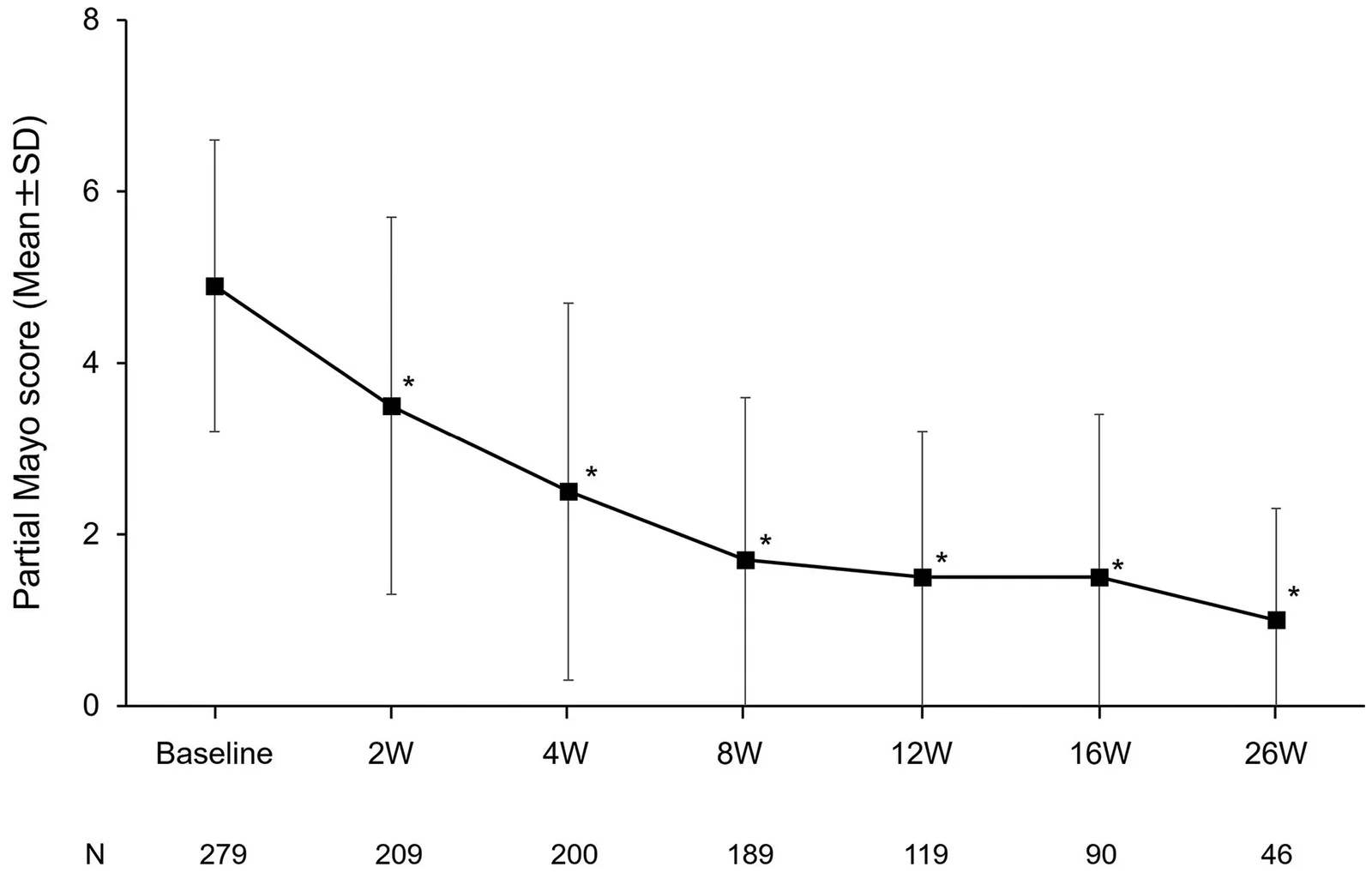
Change in partial Mayo score. *Statistically significant at *P* < .01 (Wilcoxon signed rank test vs. baseline value). A sensitivity analysis using last observation carried forward (LOCF) for missing post-baseline partial Mayo score assessments was performed as described in the Methods. Values are based on observed data available at each assessment time point. The number and composition of evaluable patients may vary across time points because treatment discontinuation and missing assessments were not imputed. W, weeks.

Figure 4A-C shows the changes in the proportions of patients with clinical response, clinical remission and rectal bleeding disappearance evaluated using the partial Mayo score and its subscores. In these 3 effectiveness assessments, the proportions of patients who achieved these endpoints increased over time from 2 weeks after the start of administration, and the proportions were 146/185 patients (78.9%), 133/189 patients (70.4%), and 137/190 patients (72.1%) after 8 weeks administration, respectively (Figure 4A-C). In a LOCF-based sensitivity analysis in which missing week-8 partial Mayo scores were imputed and endpoints were derived according to the endpoint definitions, the proportions at week 8 were 65.7% for clinical response, 55.9% for clinical remission, and 63.1% for rectal bleeding disappearance.

**Figure 4:**
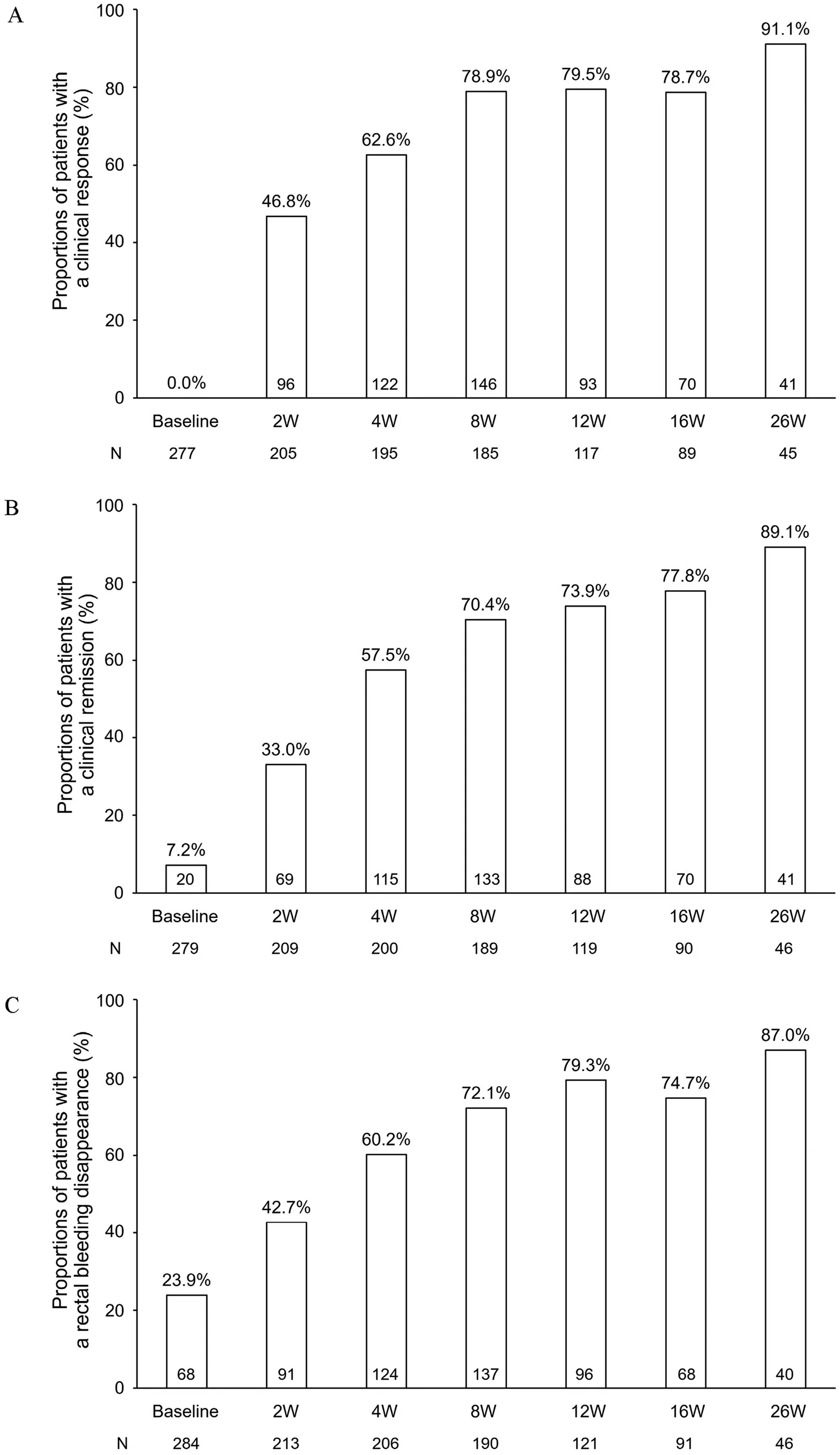
Change in the proportions of patients with a clinical response (A), clinical remission (B), and rectal bleeding disappearance (C). The number below each bar indicates a number of the patients evaluated. A last observation carried forward (LOCF)-based sensitivity analysis for week-8 endpoints is described in the Results. W, weeks.

The proportions were also evaluated at the end of administration (EOA) (at the time when each patient discontinued carotegrast administration), and the proportions were 182/276 patients (65.9%), 174/283 patients (61.5%) and 189/287 patients (65.9%), respectively. The proportions of patients with clinical response and remission were compared between subgroups stratified by the duration and disease type of UC, revealing no statistically significant differences in these comparisons.

### Other assessments

Medication adherence status was evaluated using the PRO, and the results are shown in Figure 5. In most patients (>90%), the medication adherence rate was at least 80% throughout the first-treatment phase. Figure 6 shows the PRO results for the perception of symptom improvement. The cumulative proportions of patients who perceived symptom improvement increased throughout the first-treatment phase, with a cumulative proportion of 88/190 patients (46.3%) at 2 weeks after the start of administration and 206/278 patients (74.1%) at the EOA.

**Figure 5:**
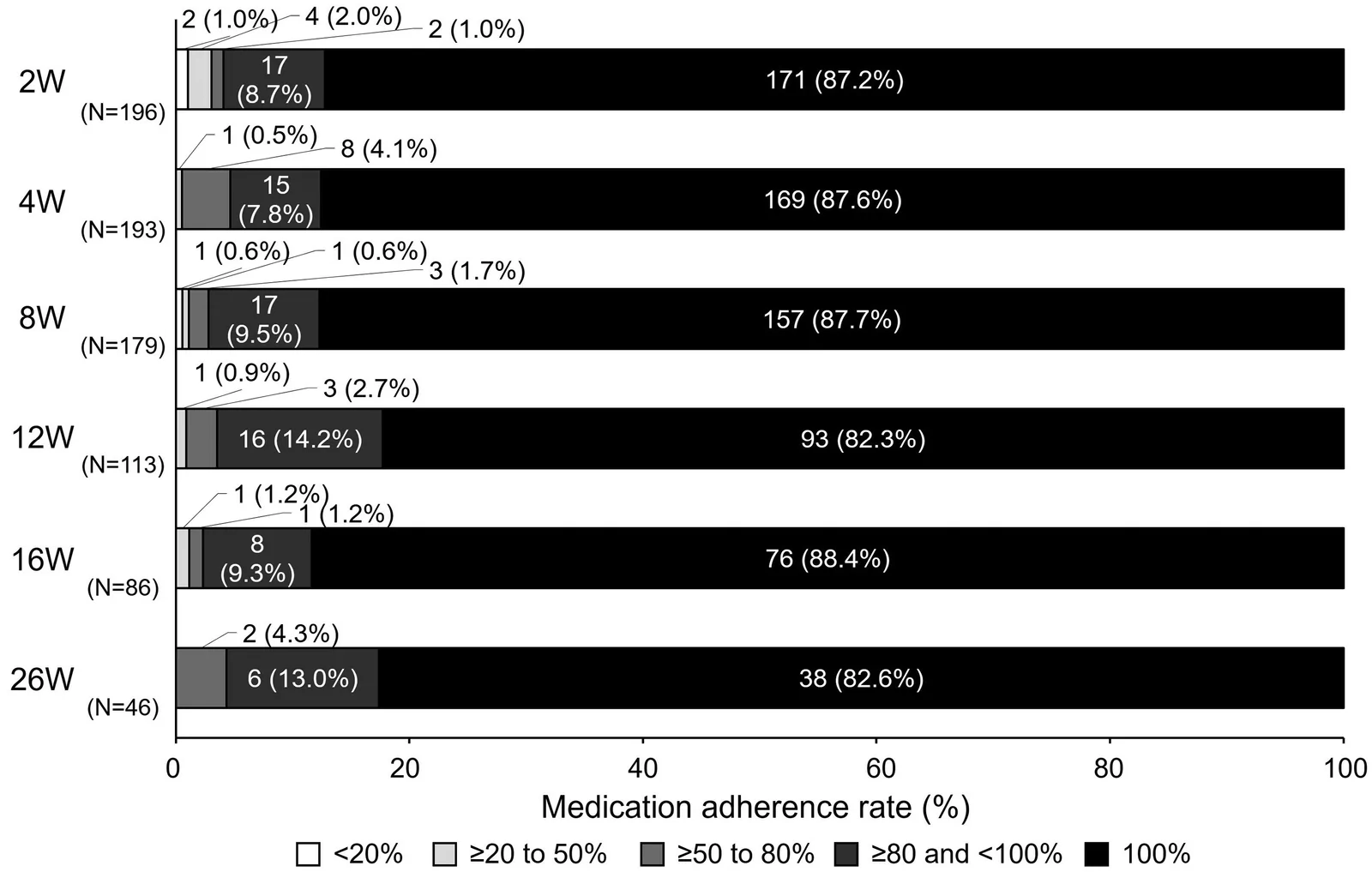
Medication adherence status. W, weeks.

**Figure 6:**
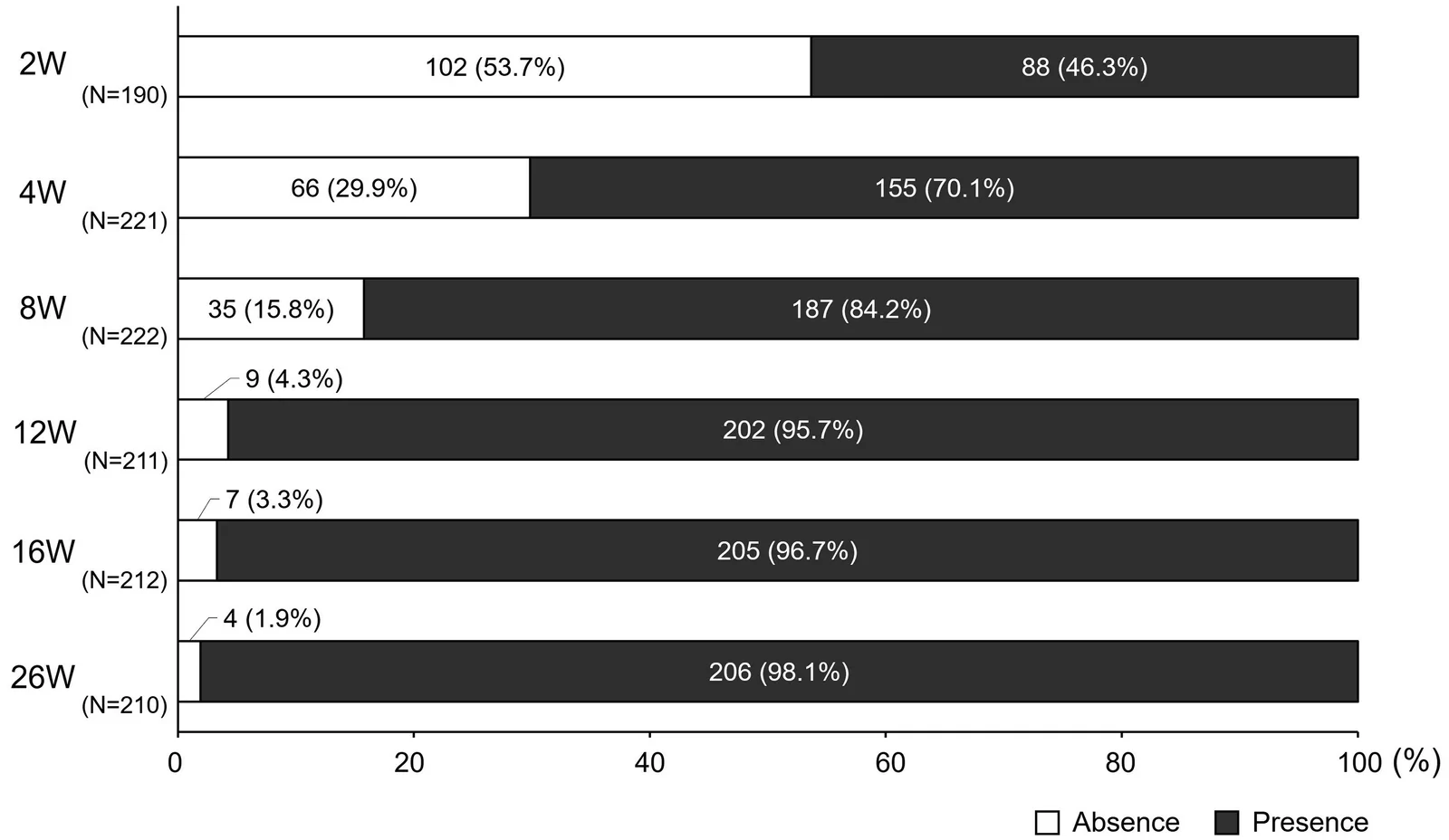
Perception of symptom improvement. W, weeks.

## Discussion

Carotegrast, a newly developed small molecule of α4-integrin antagonist, together with clinical applications of biologics, Janus kinase inhibitors, and sphingosine-1-phosphate receptor modulators, has expanded treatment options for refractory disease, UC in recent years.^3^ After the carotegrast launch in Japan in 2022, its safety and effectiveness in treating patients with UC have already been reported in 3 single center studies and 1 multicenter, retrospective study conducted in real-world settings.^11–13^ Our interim report provides the first multicenter, prospective real-world evidence of carotegrast use in Japan. These findings complement existing clinical trial data and may contribute to a better understanding of the use of carotegrast in routine clinical practice.

In the present PMS, treatment discontinuation was more commonly associated with symptom improvement than with AEs, and the overall pattern of treatment continuation and discontinuation was generally consistent with the approved use of carotegrast in Japan, in which treatment may be continued when additional therapeutic benefit is expected and discontinuation may be considered after remission is achieved. However, patients who continued carotegrast treatment beyond 8 weeks represent a selected population who did not discontinue treatment earlier because of inadequate effectiveness, disease worsening, or AEs. Therefore, these findings may be influenced by selection bias (survivorship bias), and no causal relationship between longer treatment duration and improved effectiveness can be inferred from the present observational PMS. Because UC is a chronic disease that generally requires long-term maintenance therapy, carotegrast is not positioned as a maintenance agent; rather, it is used as an induction option within the recommended treatment duration. In routine clinical practice, patients who achieve clinical remission with carotegrast may be transitioned to established maintenance therapies (eg, 5-ASA, immunomodulators, or biologics), depending on prior treatment response and individual disease characteristics.

Four patients continued carotegrast administration for >6 months, although such administration is not recommended by the prescribing information of the drug to reduce a risk of the PML occurrence.^14^ No PML was reported in these patients. However, carotegrast should not be administered over 6 months, and if needed, the drug should be administered at intervals of at least 8 weeks according to the prescribing information.^14^ In almost all patients (99.3%), the daily dose of carotegrast was administered in routine clinical practice according to the prescribing information.^14^

The incidence and pattern of ADRs observed in this PMS were generally consistent with those reported in the phase 3 study. No new safety concerns were identified in this interim analysis, and no ADRs related to PML or CNS events were observed. One patient experienced pyelonephritis and 1 patient experienced interstitial lung disease; however, no clear new safety signal emerged from these events. No pregnancies were reported during the study period. Although causality cannot be definitively established in this interim PMS, continued pharmacovigilance is warranted to monitor for PML, CNS-related ADRs, infections, and potentially unrecognized adverse reactions associated with carotegrast. Because eosinophilia may reflect either drug-related hypersensitivity or underlying inflammatory activity, clinicians should interpret eosinophil elevations alongside clinical symptoms and other inflammatory markers when assessing UC disease activity. No additional safety signals were identified among patients receiving concomitant immunomodulators; however, the subgroup size was limited, and these findings should be interpreted cautiously.

No ADRs were observed among elderly patients in this interim analysis. Japan is facing the advent of super-aging society with the percentage of people aged ≥65 years exceeding 21% of the population, and approximately 10% of newly diagnosed patients with UC are reported to be ≥65 years old according to a nationwide study.^15^ Nevertheless, there is limited high-quality evidence to provide the most appropriate management for elderly patients with IBD, as these patients are often excluded from clinical trials.^16^ Therefore, it is also important to accumulate the evidence of carotegrast use in elderly patients with UC in real-world settings to establish appropriate use of the drug for this population, although the peak age of UC onset is between 20 s and 40 s.^1^

In the effectiveness analysis set, partial Mayo scores decreased over time from 2 weeks after the start of carotegrast administration, with statistically significant improvements at all assessment time points compared with the baseline score. In the previous phase 3 study, cumulative clinical response rate on the partial Mayo score was reported to be 29% (30/102 patients) and 13% (13/101 patients) at week 2 in the carotegrast and placebo-treated group, respectively, with a statistically significant difference between the 2 groups.^8^ These data suggest that UC symptoms improved by 2 weeks after administration, and the PRO results in our analysis also showed that 88/190 patients (46.3%) perceived symptom improvement at this time. In our analysis, the partial Mayo score decreased over time up to 8 weeks after carotegrast administration, and decreased slowly thereafter. Among patients who remained on treatment, effectiveness outcomes continued to improve through Week 8. However, because patients who discontinued treatment before Week 8 were less likely to contribute data at later time points, the potential influence of selection bias should be considered when interpreting these longitudinal observations. The score at the EOA was numerically lower in patients who received carotegrast for ≥8 weeks compared with those who received it for <8 weeks. However, because treatment duration was not randomized and patients who remained on treatment beyond 8 weeks constitute a selected population, this observation should be interpreted descriptively and does not establish a causal benefit of longer treatment duration. Decisions regarding continuation of carotegrast beyond 8 weeks should be individualized, taking into account treatment response, safety, and the approved prescribing information.

Although our week-8 symptomatic outcomes appeared numerically higher, cross-study comparisons should be interpreted cautiously due to differences in study design and patient selection (routine-practice PMS vs randomized trial), as well as differences in assessment completeness and timing. In a LOCF-based sensitivity analysis that accounted for missing week-8 assessments, the corresponding rates were lower (65.7% for clinical response and 55.9% for clinical remission), suggesting that the apparent differences may be partly influenced by missing data. In addition, because the phase 3 trial’s primary endpoint used the full Mayo score including endoscopic assessment, these comparisons should be regarded as descriptive. Furthermore, the response and remission outcomes in the present PMS were defined using the partial Mayo score, which does not include endoscopic assessment. Therefore, the relatively high response and remission rates observed in this study may partly reflect differences in endpoint definitions compared with contemporary clinical trials that incorporate more stringent criteria, including endoscopic outcomes, and should not be interpreted as evidence of superior effectiveness. LOCF assumes that disease status remains unchanged after the last available assessment and may therefore overestimate or underestimate effectiveness depending on the clinical course after treatment discontinuation. The effectiveness results should be interpreted in the context of the approved treatment strategy for carotegrast in Japan. Carotegrast is used as an induction treatment rather than a maintenance therapy, and treatment discontinuation may be considered when remission is achieved. Therefore, patients who discontinued treatment after achieving a favorable clinical response do not necessarily represent treatment failures. At the same time, patients contributing effectiveness data at later assessment time points may represent a selected population because treatment duration was influenced by clinical response, safety, physician judgment, and treatment discontinuation. Consequently, both the as-observed and LOCF analyses should be interpreted descriptively, and the effectiveness results should not be regarded as estimates of outcomes in the entire treated population over time. Carotegrast, like vedolizumab, is an anti-integrin therapy that modulates lymphocyte trafficking. However, direct comparisons of effectiveness and safety between these agents are challenging because of substantial differences in treatment indications, patient populations, treatment settings, and study designs. Therefore, the present findings should be interpreted within the specific clinical context of carotegrast use in Japan.

In a previous cross-sectional web-based survey conducted in Japan, patient preference for UC therapies has been evaluated in 365 patients with UC who have a history of oral 5-ASA treatment. The survey reveals that oral administration is preferred over intravenous and subcutaneous injections in patients with mild symptoms, whereas no specific preference is observed in patients with severe symptom.^17^ In other questionnaire surveys in Japan, over 90% of patients with IBD respond that an oral formulation is favorable in their drug selection.^18–20^ Thus, oral carotegrast for moderately active UC is considered the preferred treatment option. While the regimen requires patients to take 8 tablets orally 3 times a day, some may find this high tablet burden undesirable. Despite this requirement, over 90% of patients in our study maintained ≥80% medication adherence rate, indicating a favorable adherence for carotegrast administration in real-world setting. In a previous questionnaire survey involving 485 patients with IBD, adherence to drug therapy for IBD (defined as the administration of ≥80% of the prescribed drug dose) has been investigated, revealing that 60% of patients adhere to their oral therapy such as 5-ASA.^21^ While adherence in our study was high, and may be at least comparable to previously reported adherence to oral therapies such as 5-ASA, such cross-study comparisons are limited by differences in study populations, observation periods, and adherence assessment methods.

UC is a chronic condition that requires medication throughout life, and non-adherence to medication increases the risk of clinical relapse among patients with quiescent UC.^22^ Therefore, adherence to prescribed drugs is crucial for the management of their condition. High adherence likely enhances the effectiveness of carotegrast, emphasizing the need for clinicians to clearly explain the medication’s purpose and the importance of regular dosing to each patient.

This study had some limitations. First, this was a single-arm surveillance study with no control group; therefore, the safety and effectiveness data may be influenced by confounding factors such as concomitant medications. Second, the findings were obtained from an interim analysis of an on-going PMS. This interpretation may change after the final analysis of the PMS data in the future. Third, effectiveness assessments at week 8 and later time points were affected by missing data, and the primary analysis was based on observed data without imputation. Therefore, the effectiveness analyses may be influenced by selection bias and survivor bias because patients remaining under observation at later time points may differ systematically from those who discontinued treatment earlier. Although a LOCF sensitivity analysis, including derived week-8 response and remission endpoints, supported the overall direction of effectiveness, LOCF assumes that disease activity remains unchanged after the last available assessment and may not adequately reflect the actual clinical course after treatment discontinuation. Consequently, both the as-observed and LOCF analyses should be interpreted cautiously. Fourth, the data obtained for endoscopic assessment and laboratory parameters, such as fecal calprotectin and C-reactive protein, were insufficient in this interim analysis because PMS is being conducted under routine clinical practice in Japan; thus, baseline disease characterization was limited and endoscopic/histologic remission could not be assessed, and the effectiveness results should be interpreted cautiously. Information on body composition, including visceral adiposity and skeletal muscle mass, was not collected in this PMS and was therefore unavailable for analysis. Fifth, effectiveness outcomes assessed at the EOA pooled patients who discontinued treatment due to symptom improvement and those who discontinued due to inadequate effectiveness; therefore, the EOA effectiveness rates represent a composite of heterogeneous clinical trajectories and should be interpreted with caution. In addition, because carotegrast is used as an induction treatment and discontinuation may occur after remission is achieved according to the approved prescribing information, effectiveness outcomes over time should not be interpreted in the same manner as those from studies of maintenance therapies with continuous treatment. Sixth, because this PMS was conducted in routine clinical practice in Japan, where carotegrast is currently approved and available, the generalizability of these findings to other regions may be limited. Differences in regulatory status, patient characteristics, treatment strategies, and clinical practice patterns should be considered when interpreting the applicability of these findings outside Japan. Finally, prespecified effectiveness assessments, including partial Mayo score evaluations, were conducted during the treatment period and were not routinely scheduled during the post-treatment follow-up period. Therefore, relapse rates and long-term effectiveness outcomes following treatment discontinuation could not be evaluated in the present analysis. Although the PMS includes a follow-up observation period for collection of safety information and information regarding subsequent treatment and re-treatment, the assessment of post-discontinuation disease activity is limited.

## Conclusions

In conclusion, no new safety and effectiveness concerns were found in this interim analysis for PMS with approximately 300 patients with UC treated with carotegrast in real-world clinical setting. Although further observation is required through the ongoing PMS, our current findings are based on first multicenter, prospective real-world study of carotegrast with the largest number of enrolled patients. Therefore, the study results provide useful information regarding the status of carotegrast therapy in this setting.

## Data Availability

Data are not publicly available. Deidentified data can be provided upon request to the study author.
